# Synthesis of Super-Optimized Smart Contracts Using Max-SMT

**DOI:** 10.1007/978-3-030-53288-8_10

**Published:** 2020-06-13

**Authors:** Elvira Albert, Pablo Gordillo, Albert Rubio, Maria A. Schett

**Affiliations:** 8 Microsoft Research Lab, Redmond, WA USA; 9 University of Southern California, Los Angeles, CA USA; 10 Instituto de Tecnología del Conocimiento, Madrid, Spain; 11 Complutense University of Madrid, Madrid, Spain; 12 University College London, London, U.K.

## Abstract

With the advent of smart contracts that execute on the blockchain ecosystem, a new mode of reasoning is required for developers that must pay meticulous attention to the *gas* spent by their smart contracts, as well as for optimization tools that must be capable of effectively reducing the gas required by the smart contracts. Super-optimization is a technique which attempts to find the best translation of a block of code by trying all possible sequences of instructions that produce the same result. This paper presents a novel approach for super-optimization of smart contracts based on Max-SMT which is split into two main phases: (i) the extraction of a *stack functional specification* from the basic blocks of the smart contract, which is simplified using rules that capture the semantics of the arithmetic, bit-wise, relational operations, etc. (ii) the *synthesis of optimized blocks* which, by means of an efficient Max-SMT encoding, finds the bytecode blocks with minimal gas cost whose stack functional specification is equal (modulo commutativity) to the extracted one. Our experimental results are very promising: we are able to optimize 55.41 % of the blocks, and prove that 34.28 % were already optimal, for more than 61000 blocks from the most called 2500 Ethereum contracts.

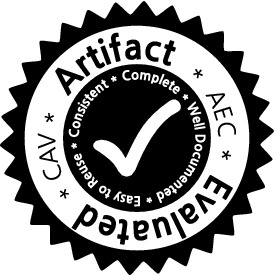



## Introduction

Open-source software that leverages on the blockchain ecosystem is known as *smart contract*. Smart contracts are not necessarily restricted to the classical concept of contracts, but can be any kind of program that executes on a blockchain or distributed ledger. A smart contract can be regarded as a collection of secured stored functions whose execution and effects (e.g., the transfer of some value between parties) cannot be manipulated. This is because all records of the transactions must be stored on a public and decentralized blockchain that avoids the pitfalls of centralization. While Bitcoin [21] paved the way for cryptocurrencies and for the popularity of the blockchain technology, Ethereum [25] showed the full potential of blockchains by allowing developers to run their decentralized applications on top of their platform. The Ethereum Virtual Machine (EVM) is capable of running smart contracts coded by Ethereum developers that have the potential of replacing all sorts of legal, financial and social agreements, e.g., can be used to fulfill employment contracts, execute bets and wagers, etc.

On the Ethereum blockchain platform, as well as in other emerging blockchains equipped with a smart contract programming language (e.g., Tezos [1], Zilliqa [24], Facebook’s Libra [23]), *gas* refers to the fee, or pricing value, required to successfully conduct a transaction or to execute a smart contract. Gas is priced in a sub-unit of the cryptocurrency—in Ethereum in *gwei*, a sub-unit of its *Ether* cryptocurrency. The EVM specification [25] provides the *gas model*, i.e., a precise definition of the gas consumption for each EVM bytecode instruction. The EVM is a simple stack-based architecture: computation on the EVM is done using a stack-based bytecode language; the word size of the machine is 256-bits (32-bytes), and this is also the size of a stack item. The proposer of a transaction allots an amount of gas (known as gas limit) to carry out the execution. If the transaction exceeds the allotted gas limit, an *out-of-gas* exception is raised, interrupting the current execution. The rationale of gas metering is three-fold: first, a gas-metered execution puts a cap on the number of operations that a transaction can execute and prevents attacks based on non-terminating executions; second, paying for gas at the moment of creating the transaction does not allow the proposer to waste other parties’ (aka *miners*) computational resources; third, gas fees discourage users to overuse replicated *storage*, which is an expensive and valuable resource in a blockchain-based consensus system.

Optimization of smart contracts has thus a clear optimization target: gas usage, as both computational and storage costs are accounted within the gas cost of each of the EVM instructions. Indeed, reducing gas costs of smart contracts is a problem of utmost relevance in the blockchain ecosystem, as there are normally between half a million and a million transactions a day. The cost of a transaction in Ethereum ranges from cents to few dollars, except in certain peak periods that has been ten or a hundred times more. In order to provide an idea of the impact of gas saving techniques, we have estimated that the money spent in transactions (excluding the intrinsic gas cost) from 2017 to 2019 is around 157 Million dollars^1^. Thus, optimizing programs in an energy-saving way is essential in general, but it is even more so in the blockchain ecosystem. The Solidity^2^ documentation [13], and posterior documents (e.g., [9, 19]), identify gas-costly patterns and propose replacements with gas-efficient ones. Adopting these guidelines requires a deep understanding of EVM instructions and the gas consumption for the different operations. Compilers for Solidity also try to optimize the bytecode for minimizing its gas consumption (e.g., the flag  of the solc compiler optimizes storage of large constants and the dispatch routine, with the goal of saving gas).

Even when the guidelines are followed and the  flag is used, the compiled EVM code is not always as efficient as desired. Super-optimization [17] is a technique proposed over 30 years ago which attempts to find the best translation of a block of code using exhaustive search to try all possible sequences of instructions that produce the same result. As an exhaustive search problem, it is computationally extremely demanding. The work in [15] proposed the idea of “unbounded” super-optimization that consists in shifting the *search* for the target program into the solver. Recently, unbounded super-optimization has been applied to Ethereum bytecode [20] for *basic block* optimization (i.e., optimizations are made inside a basic block formed by a sequence of instructions without any  operation in the middle). The experimental results in [20] confirm the extreme computational demands of the technique (e.g., the tool times out in 92% of the blocks used in their evaluation). This is a severe limitation for the use of the technique, and the problem of finding the optimal code for an EVM block still remains very challenging. The complexity stems mainly from three sources: First, the problem is expressed in the theory of bit-vector arithmetic with bit-width size of 256, which is a challenging width size for most SMT solvers. Second, expressing the problem involves an exists-forall quantification, since we want to find an assignment of instructions that works for all values in the initial stack. Third, since we look for the gas-optimal code, the problem is not a satisfaction problem but rather an optimization problem.

*Contributions.* This paper proposes a novel method for gas optimization of smart contracts which is based on synthesizing optimized EVM blocks using Max-SMT. The main novel features that distinguish our work from previous approaches, that attack the same or a similar problem [15, 20], are:

1. *Stack functional specification*. Our method takes as input an EVM bytecode and first obtains from it a stack functional specification (SFS) of the input and output operational stacks for each of the blocks of the control-flow graph (CFG) for the bytecode by using symbolic execution. The SFS determines thus the target stack that the block has to compute and is simplified using a set of rules that capture a great part of the semantics of the arithmetic, bit-wise, relational, etc., EVM operations which are relevant for gas optimization.
2. *Synthesis problem using SMT.* We approach optimization as a synthesis problem in which an SMT solver is used to synthesize optimal EVM bytecode which, for the input stack given in the functional specification, produces the target stack determined by the specification. We present a very efficient encoding that, in contrast to the previous attempts, uses only existential quantification in a very simple fragment of integer arithmetic. According to our evaluation, its simplicity greatly improves the performance of the SMT solvers while accuracy is kept as we cover the main possible optimizations. Importantly, only the semantics of the stack operations (PUSH, DUP, SWAP, etc.) is encoded, while all other operations are treated as uninterpreted functions.
3. *Use of Max-SMT*. We encode the optimization problem using Max-SMT, by adding soft constraints that encode the gas cost of the selected instructions, by adding the needed weights. This allows us to take advantage of the features given by recent Max-SMT optimizers that can improve the search.
4. *Experiments.* We report on syrup, an implementation of our approach, and evaluate it on (i) the same data set used for evaluating the tool ebso from [20] and, (ii) on 128 of the most called contracts on the Ethereum blockchain. Our results are very promising: while ebso timed out in 92.12 % of the blocks in (i), we only time out in 8.64 % and obtain gains that are two orders of magnitude larger than ebso. These results show that we have found the right balance between what is optimized by means of symbolic execution and symbolic simplification using rules and what is encoded as a Max-SMT problem. Moreover, for set (ii), we obtain gas savings of 0.59% of the total gas. Assuming that these savings are uniformly distributed, it would amount nearly to 1 Million dollars from 2017 to 2019.

While the purpose of superoptimization is to optimize at the level of basic blocks (intra-block), our approach to synthesize EVM code from a given SFS can be applied also in a richer optimization framework that enables the optimization of multiple basic blocks (inter-block). For this purpose, the framework should be extended to include branching instructions (which in the SMT encoding can be handled with uninterpreted functions as well) and, besides, additional components would be required, e.g., in the context of EVM we would need to resolve the jumping addresses, and to ensure that there are no additional incoming jumps to intermediate blocks that are being merged by the optimizer. Inter-block optimization is especially interesting in the context of smart contracts to gain storage-related gas, since the optimizations that can be achieved locally for the storage are quite limited as explained in Sect. 6.

**Fig. 1.**
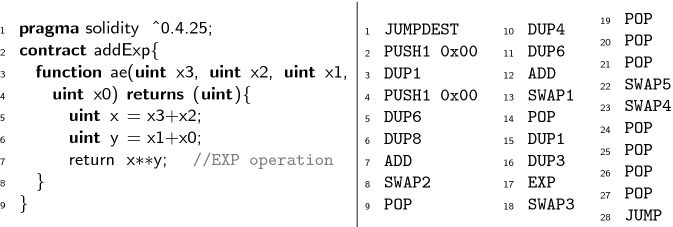
Solidity code (left). Under-optimized EVM bytecode using solc (right).

## Overview: Optimal Bytecode as a Synthesis Problem

This section provides a general overview of our method for synthesizing super-optimized smart contracts from given EVM bytecode. We use the motivating example in Fig. 1 whose Solidity source code contract appears to the left and the EVM bytecode generated by the solc compiler appears to the right. Solidity is an object-oriented, high-level language that is statically typed, supports inheritance, libraries and user-defined types, among other features. It is designed to target the EVM. As it can be observed in the example the EVM bytecodes that operate on the stack (i.e.,  ,  ,  ,  , etc.) are standard operators. In the following, we refer as *stack operations* only to  ,  ,  and  , which modify the stack without performing computations. The EVM has also bytecodes to access persistent data stored in the contract’s storage (  and  ), to access data stored in the local memory (  and  ), bytecodes that jump to a different code address location (  ,  ), bytecodes for calling a function on a different contract (  ,  ,  and  ), to write a log (  ), to access information about the blockchain and transaction (  ,  ,  , etc.) and copy information related to an external call (  ,  , etc.). However, as we explain in the coming sections, our approach is based on optimizing the operations that modify the stack as we have a great coverage of all potential bytecode optimizations while we still remain scalable, i.e., we do not optimize those bytecodes whose effects are not reflected in the stack, e.g.,  ,  ,  or  . The gas consumed by this bytecode (excluding the  and  opcodes that cannot be optimized and are thus not accounted in the examples) is 76. As specified in [25], the operations from the so-called *base* family (like  ) have cost 2, the operators from the *verylow* family (like  ,  ,  ) cost 3, operators from the *low* family (like  ,  ) cost 5, and so on.

### Extracting Stack Functional Specifications from EVM Bytecode

**Fig. 2.**
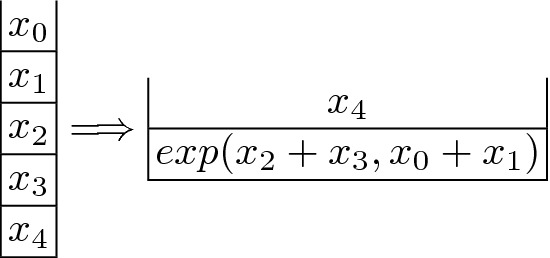
Initial and final stack

Our method takes as input the set of blocks that make up the control flow graph (CFG) of the bytecode. The first step is, for each of the blocks, to extract from it a *stack functional specification* (SFS) from which the super-optimized bytecode will be synthesized. The SFS is a functional description of the initial stack when entering the block and the final stack after executing the block, which instead of using bytecode instructions to determine how the final stack is computed, is defined by means of *symbolic first-order terms* over the initial stack elements. The SFS for our running example is shown in Fig. 2.

As can be observed, it consists of an initial stack shown at the left which simply determines what the size of the input stack to the block is and assigns a symbolic variable as identifier to each stack position (e.g., the initial stack contains five elements named $x_0,\ldots ,x_4$); while the output stack contains two elements: $x_4$ at the top, and the symbolic term $exp(x_2+x_3,x_0+x_1)$ at the bottom. The output stack is obtained by symbolic execution of the bytecodes that operate on the stack, as it will be formalized in Sect. 3. The resulting expressions are then optimized by means of simplification rules based on the semantics of the non-stack operations (e.g., the neutral elements, double negations or idempotent operations are removed, operations on constants performed). This captures a relevant part of the semantics of the non-stack operators.

### The Synthesis Problem

This section hints on how the generated bytecode will be, and on that the synthesis of optimal bytecode from the specification is challenging.

#### Example 1

From the SFS in Fig. 2, we know that we have to compute $x_0+x_1$ and $x_2+x_3$, but we have to decide which summation we compute first. On the left, we have the best bytecode (together with the stack evolution) when we first compute $x_2+x_3$ and on the right when we first compute $x_0+x_1$. Computing first one subexpression or the other has an impact on the consumed gas, since the bytecode on the left has a gas cost of 31 and the bytecode on the right has a gas cost of 25, which is indeed the optimum.


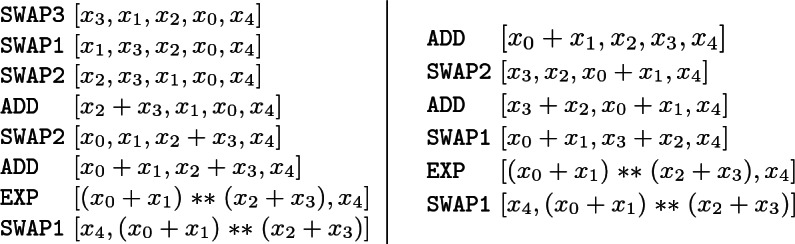


Both codes are far better than the original generated bytecode whose gas cost was 76. Besides, note that the cost of the two additions and the exponentiation is in total 16 (that necessarily has to remain), which means that the optimal code has used only 9 units of gas for the rest while the original code needed 60 units.

The next example shows that the optimal code is obtained when the subterms of the exponential are computed in the other order (compared to the previous example). Hence, an exhaustive search of all possibilities (with its associated computational demands) must be carried out to find the optimum.

#### Example 2

Let us now consider a slight variation of the previous example in which the functional specification is $$[x_0,x_1,x_2,x_3]\Longrightarrow [x_3,(x_0+x_1)\mathrel {**}(x_0+x_2)]$$. Now, on the left-hand side we have the best bytecode (together with the stack evolution) when we compute first $$x_0+x_2$$ and on the right-hand side we have the best bytecode when we compute first $$x_0+x_1$$.


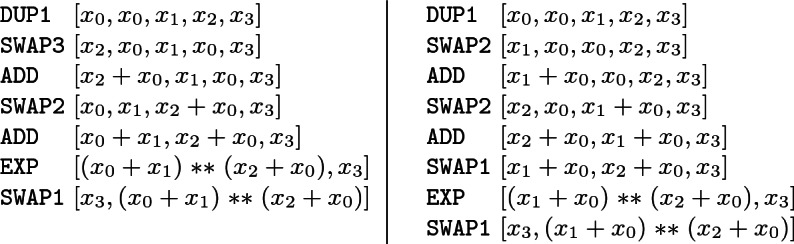


In this case the bytecode on the left has a gas cost of 28, which is indeed the optimum, and the bytecode on the right has a gas cost of 31. The original bytecode generated by solc has gas cost 74, so again the improvement is huge.

Both examples show that, in principle, even if we have the functional specification that guides the search, we have to exhaustively try all possible ways to obtain it, if we want to ensure that we have found the optimal bytecode.

### Characteristics of Our SMT Encoding of the Synthesis Problem

Our approach to super-optimize blocks is based on restricting the problem in such a way that we have both a great coverage of most EVM code optimizations and we can propose an encoding in a simple theory where an SMT solver can perform efficiently. To this end, the key point is to handle all non-stack operations, like  ,  ,  ,  ,  , as *uninterpreted bytecodes*. This allows us to simplify the encoding in two directions. First, by considering them as uninterpreted bytecodes we can avoid reasoning on the theory of bit-vectors with width 256. Second, and even more important, this allows us to express the problem in the existentially quantified fragment, avoiding the exists/forall alternation:

1. We start from the SFS by introducing fresh variables abstracting out all terms built with uninterpreted functions, in such a way that every fresh variable represents a term $$f(a_1,\ldots ,a_n)$$, where every $$a_i$$ is either a (256 bit) numeric value, a fresh variable, or an initial stack variable. We also have sharing by having a single variable for every term, e.g., $$(x_0+1)\mathrel {**}(x_0+1)$$, where $$x_0$$ is the top of the initial stack, is abstracted into $$y_0=$$  $$_\mathtt{U}(y_1,y_1)$$ and $$y_1=$$  $$_\mathtt{U}(x_0,1)$$, where $$y_0$$ and $$y_1$$ are fresh variables and  $$_\mathtt{U}$$ and  $$_\mathtt{U}$$ are the uninterpreted bytecodes for exponentiation and addition, respectively.
2. Now, in order to avoid universal quantification, we take advantage of the fact that only values from 0 to $2^{256}-1$ can be introduced in the stack by a  opcode and hence only this range can appear in the SFS. Therefore, if we assign values from $2^{256}$ on to fresh variables and initial stack variables we avoid the confusion between themselves and all other values in the problem.

After these two key observations have been made, we fix the maximal number *n* of opcodes and highest size *h* of the stack that is allowed in a solution. This can be bound by analyzing the original code generated by the compiler. From this, we roughly encode the problem using variables $o_0,\ldots ,o_{n-1}$ to express the operations of our code (together with variables $p_0,\ldots ,p_{n-1}$ that encode the value $0\le p_i \le {2^{256} - 1}$ added to the stack when $o_i$ is a  ), variables $s^i_0,\ldots ,s^i_{h-1}$ to encode the contents of the stack before executing the operation $o_i$, where $s^i_0$ is the top of the stack (we also use some Boolean variables to express the active part of the stack). Using this, we can encode the behavior of all stack operations:  ,  ,  ,  for all its versions (like  ,  , ...). For the uninterpreted bytecodes $f_u$, we basically add for every abstraction $y=f_u(a_1,\ldots ,a_m)$ assertions stating that if we have $a_1,\ldots ,a_m$ at the top of the stack at step *i* (i.e., $s^i_0,\ldots ,s^i_{m-1}$) and we take the operation *f* in $o_i$ then in step $i+1$ we have $y,s^i_m,\ldots$ on the top of the stack. Again, as all fresh variables and initial stack variables have been replaced by values form $2^{256}$ on, there is no confusion with all other values.

As a final remark, we have also encoded the commutativity property of uninterpreted bytecodes representing the  ,  ,  ,  , etc. This can be easily made by considering that the arguments can occur at the top of the stack in the two possible orders. Other properties like associativity are more difficult to encode and are left for future developments.

### Optimal Synthesis Using Max-SMT

The last key element is how we encode the optimization problem of finding the bytecode with minimal gas cost. First, let us describe which notion of optimality we are considering. Our problem is defined as, given an SFS in which all occurring bytecodes there are considered uninterpreted and maybe commutative, we have to provide the bytecode with minimal gas cost whose SFS is equal modulo commutativity to the given one. From the encoding we have described in the previous section, we know that every solution to the SMT problem will have the same SFS as the given one. Hence, we only need to find the solution with minimal gas cost. In [20], this was made by implementing a loop on top of the SMT solving process which was calling the solver asking every time for a better solution in terms of gas, which was also encoded in the SMT problem. Such approach cannot be easily implemented in an incremental way using the SMT solver as a black box without the corresponding performance penalty.

Alternatively, we propose to encode the problem as a Max-SMT problem and hence, we can easily use any Max-SMT optimizer, like Z3 [12], Barcelogic [7] or (Opti)MathSAT [11], as a black box with an important gain in efficiency. The Max-SMT encoding adds to the previously defined SMT encoding some soft constraints, indicating which is the cost associated to choosing every family of operators. As mentioned, choosing an operator from the *base* family has cost 2, from the *verylow* 3, and so on. Then, the optimal solution is the solution that minimizes this cost, which can be obtained with a Max-SMT optimizer.

**Fig. 3.**
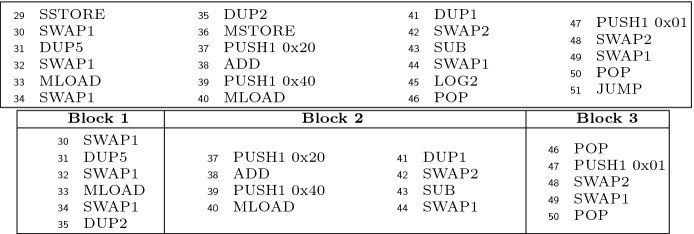
CFG block of a real smart contract (top), and blocks generated to build the functional description of the EVM bytecode (bottom)

## Stack Functional Specification from EVM Bytecode

The starting point of our work is the CFG of the EVM bytecode to be optimized. There are already a number of tools (e.g., EthIR [6], Madmax [14], Mythril [18] or Rattle [4]) that are able to compute the CFG from the bytecode of a given smart contract. Therefore, we do not need to formalize, neither to implement, this initial CFG generation step. Since there are bytecode instructions that we do not optimize, for each of the blocks of the provided CFG, we first perform a further block-partitioning that splits a basic block into the sub-blocks that will be optimized by our method as defined below. A basic block is defined as a sequence of EVM instructions without any  bytecode.

### Definition 1 (block-partitioning)

Given a basic block $B =[b_0,b_1,...,b_n]$, we define its block-partitioning as follows:where


As it can be observed, the bytecodes whose effects are not reflected on the stack induce the partitioning and are omitted in the fragmented sub-blocks. These include the bytecodes that modify the memory, the storage or record a log, that belong to the *Split* set. Figure 3 shows a CFG block at the top and the blocks generated to build the functional description at the bottom. The original CFG block contains the bytecodes  ,  and  . Thus, it is split into three different blocks that do not contain these bytecodes.

Once we have the partitioned blocks from the CFG, we aim at obtaining a functional description of the output stack (i.e., the stack after executing the sequence of bytecodes in the block) using symbolic execution for each of the partitioned blocks. As the stack is empty before executing a transaction and the number of elements that each EVM bytecode consumes and produces is known, the size of the stack at the beginning of each block can be inferred statically. We can thus assume that the initial stack size is given within the CFG. A symbolic stack $\mathcal {S}$ is a list of size *k* that represents the state of the stack where the list position 0 corresponds to the top of the stack and $k-1$ is the index of the bottom of the stack, such that $\mathcal{S}[i]$ is the symbolic value stored at the position *i* of the stack. Initially, the input stack maps each index to a symbolic variable $s_i$.

**Fig. 4.**
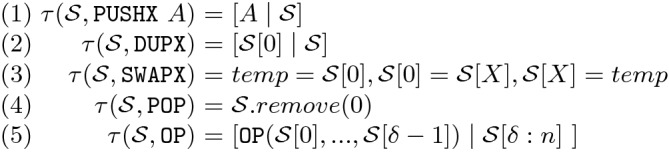
Symbolic execution of the instructions that operate on the stack

The symbolic execution of each bytecode is defined using the transfer function $\tau$ described in Fig. 4 which takes an input stack and a bytecode and returns the output stack as follows: (1) the  bytecode stores at the top of the stack the value *A*, (2)  duplicates the element stored at position  $-1$ to the top of the stack, (3)  exchanges the values stored at the top of the stack with the one stored at position  , (4)  deletes the value stored in the top of the stack (using the list operation *remove* to delete the element at the given position), (5)  represents all other EVM bytecodes that operate with the stack (arithmetic and bit-wise operations among others). In that case, $\tau$ creates a symbolic expression that is a functor with the same name as the original EVM bytecode and as arguments the symbolic expressions stored in the stack elements that it consumes. Here, $\delta$ stands for the number of elements that the EVM bytecode  gets from the stack. Now, the SFS can be defined using the function $\tau$ as follows.

### Definition 2 (SFS)

Given a block *B* with an initial size of the stack *k*, the initial state of the stack $\mathcal{S}_0$ stores at each position $i \in \{0,...,k-1\}$ a symbolic variable $s_i$. Then, the transfer function $\tau$ is extended to the block *B*, denoted by $\tau (B)$, as: $[s_0,\ldots ,s_{k-1}]$ if *B* is empty; and $\tau (\tau (B'),o)$ if *B* has *o* as last operation and $B'$ is the resulting block without *o*. The SFS of *B* is $\mathcal{S}_0$ $\Longrightarrow$ $\mathcal{S}$ $=\tau (B)$.

### Example 3

Consider the block formed by the EVM bytecode shown in Fig. 1, starting with the bytecode at program point 2 (pp2 for short) and finishing with the bytecode at pp27. Before executing the block symbolically, the initial stack is $\mathcal{S}_0 =[s_0,s_1,s_2,s_3,s_4]$ and $k=5$. After applying the transfer function $\tau$, we obtain the following results at the next selected program points:


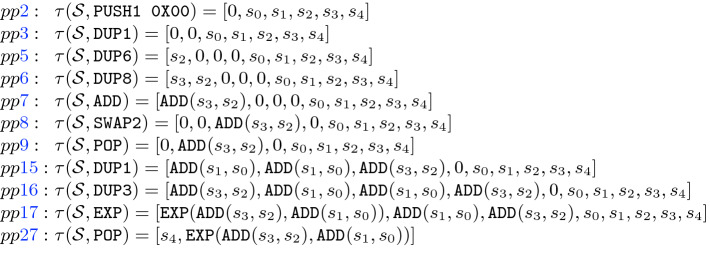


Thus, altogether, the output stack of the SFS given by $\tau$ for the block in Fig. 1 is . For example, we can see that $\tau$ updates the stack inserting a 0 in the top of the stack at pp2. At pp8, it swaps the element in the top of the stack () with the element stored at position 2 (0). It generates a symbolic expression to represent the addition at pp7 with the values stored in the position of the stack that it consumes. At pp17 it generates a new symbolic expression  to represent the exponentiation of the two elements stored in the top of the stack. Note that in this case these elements are also symbolic expressions of the two previous additions symbolically executed before.

Finally, we capture optimizations based on the semantics of the arithmetic and bit-wise operations, by applying simplification rules on the SFS of the block before we proceed to generate the optimized code. This simplification besides reducing the number of operations includes other notions of simplification as well. The easiest examples are the application of simplification rules like with the units of every operation, or with the idempotence of bit-wise Boolean operators.

## Optimal Synthesis Using Max-SMT

This section describes our Max-SMT encoding. We start by preprocessing the SFS into an abstract form that is convenient for the encoding in Sect. 4.1. Next, Sect. 4.2 describes a key element of our encoding: the stack model. Sect. 4.3 presents the complete encoding of the problem and Sect. 4.4 how to obtain the optimized EVM blocks from the model obtained by the SMT solver. Finally, Sect. 4.5 describes the optimization problem. The SFS and the encoding generated for the example shown in Fig. 1 are available at https://github.com/mariaschett/syrup-backend/tree/master/examples/cav2020.

### Abstracting Uninterpreted Functions

Before we apply our encoding, we need to abstract all (sub)expressions occurring in the SFS, by introducing new fresh variables $s_k,s_{k+1},\ldots$ that start after the last stack variable in the initial stack $[s_0,\ldots ,s_{k-1}]$ (of size *k*). In this process we have a mapping from fresh variables to shallow expressions of depth one, i.e., built with a function symbol and variables or constants as arguments. Here we introduce the *minimal* number of fresh variables that allow us to describe the SFS using only shallow expressions. By minimal, we mean that we use the same variable if some subterm occurs more than once (we also take into account commutativity properties to avoid creating unnecessary fresh variables). Finally if an uninterpreted function occurs more than once, we add a subscript from 0 on to distinguish them. As a result we have that the *abstracted SFS* is defined by a stack *S* containing only stack variables, fresh variables or constants (in $\{0,\ldots ,2^{256}-1\}$) and a map *M* from fresh variables to shallow terms formed by an uninterpreted function (maybe with subscript) applied to stack variables, fresh variables or constants (in $\{0,\ldots ,2^{256}-1\}$). Besides, we note that the *abstracted SFS* generated is equivalent to first-order A-normal form with shearing. Trivially, all positions in the stack in the SFS and the abstracted SFS are equal when the map is fully applied to remove all fresh variables and the subscripts are removed. Moreover, we have that every uninterpreted function of the SFS has a fresh variable assigned in the map and all function symbols in the map are different.

#### Example 4

The abstraction of the SFS  shown in Example 3 needs three fresh variables $s_5$, $s_6$ and $s_7$. Then, the abstracted SFS is the stack $S=[s_4,s_7]$ and the mapping *M* is defined as .

### Modeling the Stack

A key element in our encoding is the representation of the stack and the elements it contains. As mentioned in Sect. 2.3, a first observation is that in our approach we will only have in the stack constants in the domain $\{0,\ldots ,2^{256}-1\}$ (we do not care if they represent a negative number or not, as they are handled simply as 256-bit words), initial stack variables $s_0,\ldots ,s_{k-1}$ and fresh variables $s_k,\ldots ,s_v$. In order to distinguish between constants and the variables $s_i$, we assign to every variable $s_i$, with $i\in \{0,\ldots ,v\}$, the constant $2^{256}+i$. Now, for instance, we can establish that a  operation can only introduce a constant in $\{0,\ldots ,2^{256}-1\}$ and that fresh variables $s_i$ can only be introduced by uninterpreted functions if the appropriate arguments are in the stack (see below). The rest of stack operations, like  or  , just duplicate or move whatever is in the stack. Since in our encoding we will use the variables $s_0, \ldots , s_v$, as they are part of the SFS, we have a first constraint assigning the constant values to all these variables (this could be done as well with a *let* expression).

$$\begin{aligned} S_V = {\bigwedge \nolimits _{0 \leqslant i < v} {s_i = 2^{256} + i}} \end{aligned}$$

Let us now show how we model the stack along the execution of the instructions. First, we have to fix a bound on the number of operations $b_o$ and the size of the stack $b_s$. We can apply different heuristics to this end though considering the initial number of operations and the maximum number of stack elements involved in the block are sound bounds. We have to express a stack of $b_s$ positions after executing *j* operations with $j\in \{0,\ldots ,b_o\}$. To this end, on the one hand, we use existentially quantified variables $x_{i,j}\in \mathbb {Z}$ with $i\in \{0,\ldots ,b_s-1\}$ and $j\in \{0,\ldots ,b_o\}$ to express the word at position *i* of the stack after executing the first *j* operations of the code, where $x_{0, j}$ encodes the word on the top of the stack. On the other hand to complete the modeling we introduce propositional variables $u_{i,j}$ with $i\in \{0,\ldots ,b_s-1\}$ and $j\in \{0,\ldots ,b_o\}$, to denote the *utilization* of the stack (i.e., the words that the stack currently holds). Here, $u_{i,j}$ indicates that the word at position *i* of the stack after executing the first *j* operations exists or not.

Additionally, to simplify the next definitions we have the following parameterized constraint that, given an instruction step *j* with $0 < j \le b_o$, two stack positions $\alpha$ and $\beta$ and a shift amount $\delta \in \mathbb {Z}$, with $0\le \alpha$, $0\le \alpha +\delta$, $\beta <b_s$ and $\beta +\delta <b_s$, imposes that the stack after executing $j+1$ instructions between positions $\alpha$ and $\beta$ is the same as the stack after executing the *j* instruction but with a shift of $\delta$ (they are moved up if negative and moved down otherwise).

$$\begin{aligned} Move(j,\alpha ,\beta ,\delta ) = {\bigwedge \nolimits _{\alpha \leqslant i \leqslant \beta } \; u_{i+\delta ,j+1} = u_{i,j} \;\wedge \; x_{i+\delta ,j+1} = x_{i,j}} \end{aligned}$$

### Encoding of Instructions

Let $\mathcal {I}$ be the set of instructions occurring in our problem. The set $\mathcal {I}$ is split in three subsets $\mathcal {I}_C \uplus \mathcal {I}_U \uplus \mathcal {I}_S$, where:

- $\mathcal {I}_C$ contains the commutative uninterpreted functions occurring in the map *M* of the abstracted SFS,
- $\mathcal {I}_U$ contains the non-commutative uninterpreted functions occurring in *M*,
- $\mathcal {I}_S$ contains the stack operations:  , that introduces an up to 32-bytes item on top of the stack;  that removes the top of the stack; *k*, with $k\in \{1,\ldots , 16\}$ that copies the $k{-}1$ element of the stack on top of the stack; *k*, with $k\in \{1,\ldots ,16\}$ that swaps the top of the stack with the *k* element of the stack; and an extra operation  that does nothing.

Note that, although in EVM there are 32 different  instructions depending on the amount of bytes needed to express the item, in our context this distinction is unnecessary, since we can decide afterwards which  do we need by checking in the obtained solution which is the value to be pushed. Also, the operations *k* in $\mathcal {I}_S$ are reduced to only those with $k<b_s$ (otherwise we go beyond the maximal size of the stack) and, similarly, the operations *k* in $\mathcal {I}_S$ are reduced to only those with $k<b_s$.

Let $\theta$ be a mapping from the set of instructions in $\mathcal {I}$ to consecutive different non-negative integers in $\{0,\ldots ,m_\iota \}$, where $m_\iota +1$ is the cardinality of $\mathcal {I}$. In order to encode the selected instructions at every step, we introduce the existentially quantified variables $t_j\in \{0,\ldots ,m_\iota \}$, with $j\in \{0,\ldots ,b_o-1\}$ where for every instruction $\iota \in \mathcal {I}$, if $t_j=\theta (\iota )$ then we have that the operation executed at step *j* is $\iota$. Additionally, we introduce associated existentially quantified variables $a_j\in \{0,\ldots ,2^{256}-1\}$, with $j\in \{0,\ldots ,b_o-1\}$, to express the value pushed at the top of the stack when $t_j=\theta ($  ) (otherwise the value of $a_j$ is meaningless).

**Encoding the Stack Operations.** First we show how we encode the effect of choosing in $t_j$ one of the operations in $\mathcal {I}_S$ that does not depend on the particular (abstracted) SFS we are considering. The following parameterized constraints show this effect:
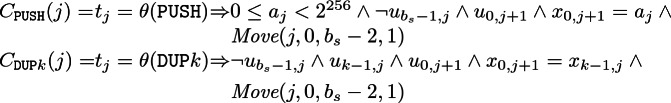
 
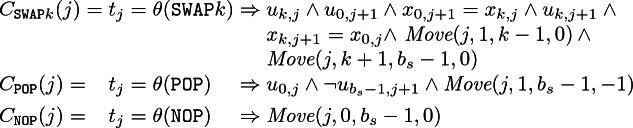


Notice that the stack before executing the instruction $t_j$ is given in the variables $x_{0,j},\ldots ,x_{b_s-1,j}$ and $u_{0,j},\ldots ,u_{b_s-1,j}$, while the stack after executing $t_j$ is given in $x_{0,j+1},\ldots ,x_{b_s-1,j+1}$ and $u_{0,j+1},\ldots ,u_{b_s-1,j+1}$.

In order to avoid redundant solutions (with  in intermediate steps), we have to add as well a constraint stating that once we choose  as instruction $t_j$ we can only choose  for the following instructions $t_{j+1}, t_{j+2} \ldots$:

$$\begin{aligned} C_{\texttt {fromNOP}} = {\bigwedge \nolimits _{0 \leqslant j < b_o-1} \; t_j =\theta (\texttt {NOP}) \Rightarrow t_{j+1} =\theta (\texttt {NOP})} \end{aligned}$$

**Encoding the Uninterpreted Operations.** The encoding of the uninterpreted operations comes from the map *M* of the abstracted SFS. First of all, note that, every function *f* occurs only once in *M* (since subscripts are introduced) and for every $r\mapsto f(o_0,\ldots ,o_{n-1})$ in *M* we have that $f\in I_C\uplus \mathcal {I}_U$, *r* is a fresh variable, and $o_0,\ldots ,o_{n-1}$ are either initial stack variables, fresh variables or constants. Note also that if $f\in \mathcal {I}_C$ then $n=2$. Therefore, we define in the encoding the effect of choosing in $t_j$ the uninterpreted function *f* with $r\mapsto f(o_0,\ldots ,o_{n-1})$ in *M*, as an operation that takes its arguments $o_0,\ldots ,o_{n-1}$ from the stack and places its result *r* in the stack (where $o_0$ must be at the top of the stack).
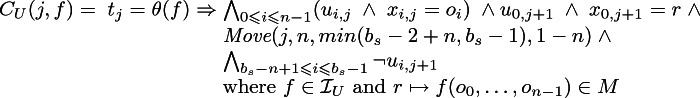


Now for the commutative functions the only difference is that we know that $n=2$ and that we can find the arguments in any of both orders in the stack:
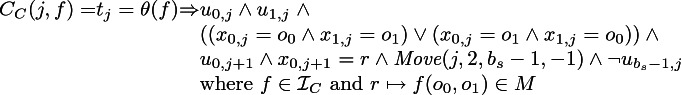
**Finding the Target Program.** We assign to every ${\iota \in \mathcal {I}}$ an integer. Then, ${t_j \in \mathbb {Z}}$ encodes the chosen instruction at position *j* in the target program for ${0 \leqslant j < b_o}$. To encode the selection of an instruction for every $t_j$, we have the following constraint:
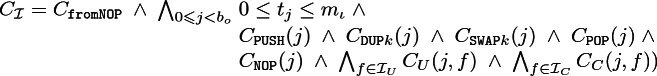


**Complete Encoding.** Let us conclude our encoding by defining the formula $C_{SFS}$ that states the whole problem of finding an EVM block for a given initial stack $[s_0, \ldots , s_{k-1}]$ and abstracted SFS with final stack $[f_0,\ldots ,f_{w-1}]$ and map *M*. Hence, we introduce a constraint *B* to describe how the stack at the beginning is and a constraint *E* to describe how the stack at the end is and combine all the constraints defined above to express $C_{SFS}$.
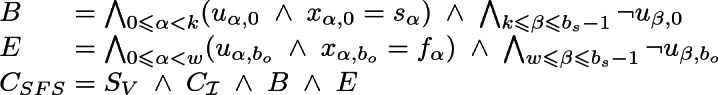


Finally, let us mention that the performance of the used SMT solvers greatly improves when the following (redundant) constraint, which states that all functions in $\mathcal {I}_U\uplus \mathcal {I}_C$ should be eventually used, is added: $\bigwedge _{\iota \in \mathcal {I}_U\uplus \mathcal {I}_C} \bigvee _{0 \leqslant j < b_o} t_j = \theta (\iota )$

Empirical evidence shows, that this constraint helps the solver to establish optimality, and removing it increases the time-outs and time taken by roughly 50%. On the other hand, adding the similar constraint that all functions in $\mathcal {I}_U\uplus \mathcal {I}_C$ are used at most once, while also helping the solvers to show optimality for already optimal blocks, the performance for finding optimizations decreases by a similar rate. As the latter is our main motivation, we did not include the constraint.

### From Models to EVM Blocks

The following definition shows how we can extract a concrete set of operations from a model for the formula $C_{SFS}$ that computes the given SFS.

#### Definition 3

Given a model $\sigma$ for $C_{SFS}$ we have that $block(\sigma )$ is defined as the sequence of EVM operations $o_0,\ldots ,o_f$ where *f* is the largest $j\in \{0,\ldots ,b_o-1\}$ such that $t_j\not =\theta (\texttt {NOP})$. Now for all $\alpha \in \{0,\ldots , f\}$ the operation $o_\alpha$ is taken as

1. $o_\alpha = \texttt {PUSH}k \; a_\alpha$ if $t_\alpha = \theta (\texttt {PUSH})$ and $a_\alpha$ can be represented with *k* bytes.
2. $$o_\alpha = \iota $$ if $$t_\alpha = \theta (\iota )$$ where $$\iota \in \mathcal {I}_S\setminus \{\texttt {PUSH}\}$$
3. $o_\alpha = \iota$ if $t_\alpha = \theta (\iota )$ where $\iota \in {\mathcal {I}_U\uplus \mathcal {I}_C}$ and $\iota$ has no subscript.
4. $o_\alpha = \iota$ if $t_\alpha = \theta (\iota _l)$ where $\iota _l\in {\mathcal {I}_U\uplus \mathcal {I}_C}$ and has subscript *l*.

The following result easily follows from the construction of $C_{SFS}$.

#### Theorem 1 (soundness)

Given an SFS and values for $b_o$ and $b_s$, we have that if $\sigma$ is a model for $C_{SFS}$ obtained from the abstracted SFS then $block(\sigma )$ computes the given SFS.

### Optimization Using Max-SMT

Now that we know that every model of $C_{SFS}$ provides a block that computes the SFS, we want to obtain the optimal solution. Since the cost of the solution can be expressed in terms of the cost of every of the instructions we select in all $t_j$, we will introduce soft constraints expressing the cost of every selection. A (partial weighted) Max-SMT problem is an optimization problem where we have an SMT formula which establishes the *hard constraints* of the problem and a set of pairs $\{ [C_1,\omega _1], \ldots ,[C_m,\omega _m] \}$, where each $C_i$ is an SMT clause and $\omega _i$ is its weight, that establishes the *soft constraints*. In this context, the optimization problem consists in finding the model that satisfies the hard constraints and minimizes the sum of the weights of the falsified soft constraints. Our approach to find the optimal code is by encoding the problem as a Max-SMT optimization problem, where we add to the SMT formula $C_{SFS}$ which defines our *hard constraints* a set of *soft constraints* such that sum of the weights of the falsified soft constraints coincides with the cost (in terms of gas) of the operations taken in every step. Therefore the optimal solution to the Max-SMT problem coincides with the optimal solution in terms of gas cost.

In the EVM, every operation has an associated gas cost, which in general is constant, but in some few cases may depend on the particular arguments it is applied to or on the state of the blockchain. All these operations that are non-constant are considered as uninterpreted, and hence we cannot change the operands on which they are applied. Therefore, omitting the non-constant part cannot affect which is the optimal solution. Thanks to this, we can split our set of instructions $\mathcal {I}$ in $p+1$ disjoint sets $W_0\uplus \ldots \uplus W_p$ where all instructions in $W_i$ have the same constant cost $\mathsf {cost}_i$, and such that the costs are strictly increasing, i.e., $\mathsf {cost}_0 = 0$ and $\mathsf {cost}_{i-1} < \mathsf {cost}_{i}$ for all $i\in \{1,\ldots , p\}$.

In the following we describe the encoding we have chosen for the weighted clauses (we have tried other slightly simpler alternatives but, in general, they behave worse). Let $w_i = \mathsf {cost}_{i}-\mathsf {cost}_{i-1}$ for $i\in \{1,\ldots , p\}$. Hence, we have that $w_i>0$ and, moreover, $\mathsf {cost}_{i}=\varSigma _{1 \leqslant \alpha \leqslant i}w_\alpha$ for $i\in \{1,\ldots , p\}$. Then, our Max-SMT problem $O_{SFS}$ is obtained adding to $C_{SFS}$ the following soft constraintsTherefore, if the selected instruction at step *j* is $\iota$ (i.e. $t_j=\theta (\iota )$) for some $\iota \in W_i$ then we accumulate the weight $w_\alpha$ of all soft clauses with $\alpha \in \{1,\ldots , i\}$, which as said sums $\mathsf {cost}_{i}$, and hence we accumulate the cost of executing the instruction $\iota$. From this fact, our optimality theorem follows.

#### Theorem 2 (optimality)

Given an SFS *P* and values for $b_o$ and $b_s$, we have that if $\sigma$ is the optimal solution for the weighted Max-SMT problem $O_{SFS}$ obtained from the abstracted SFS of *P*, then $block(\sigma )$ is the optimal code that has an SFS equal to *P* modulo commutativity.

## Experimental Evaluation

This section presents the results of our evaluation using syrup, the SYnthesizeR of sUPer-optimized smart contracts that implements our approach. Our tool syrup uses EthIR [6] to generate the CFGs of the analyzed contracts and Z3 [12] version 4.8.7, Barcelogic [7], and MathSAT [11] version 1.6.3 (namely its optimality framework OptiMathSAT), as SMT solvers. We refer by s-Z3, s-Bar, s-OMS, to the results of using syrup with the respective solvers. Experiments have been performed on a cluster with Intel Xeon Gold 6126 CPUs at 2.60 GHz, 2 GB of memory and timeout of 15 min, running CentOS Linux 7.6. The main components of syrup are implemented in Python and OCaml. The backend of syrup generating SMT constraints from a SFS is open-source and can be found at github.com/mariaschett/syrup-backend. Our tool accepts smart contracts written in versions of Solidity up to 0.4.25 and EVM bytecode v1.8.18, namely the three new EVM bytecodes (  ,  and  ) introduced from the Solidity compiler version 0.5.0 are not handled yet by EthIR. Our experimental setup consists of two groups of benchmarks:

(i) In order to compare with the existing tool ebso, we use the same data set (and the results for ebso) from [20]: the blocks of the 2500 most called contracts deployed on the Ethereum blockchain^3^ after removing the duplicates and the blocks which are only different in the arguments of  by abstracting to word size 4 bit. This results in a data set of 61217  blocks.
(ii) A more realistic setting in which we analyze the 150 most called contracts^4^ queried from the Ethereum blockchain and removing those of the versions not supported, resulting in 128. As the dates in which the contracts are fetched are different, not all 128 contracts are included in setup (i), indeed, the intersection are 106 contracts (besides there might be updated versions). This setting is more realistic since the analysis is performed at the contract-level (without removing any duplicates or similar blocks) and allows us to gather statistics to assess the gains at the level of the deployed contracts.

We note that analyzing the most called contracts corresponds to the most relevant case study as, according to [16], many Ethereum contracts are not used.

### Comparison with ebso (setup I)

As seen in Definition. 1, we split the 61217  blocks on certain bytecodes that are not optimized, leading to a total of 72450. For comparison, we merge the split blocks back together. The next table shows the results of optimizing the 61217 blocks by ebso (first column), and by syrup for every solver (next columns). In column s-All, we use the 3 solvers as a single framework in syrup that yields the best solution returned by any of the solvers (in parenthesis we show percentages).

**Table:** 

	ebso	s-Z3	s-Bar	s-OMS	s-All
A	3882 (6.34%)	20636 (33.71%)	20783 (33.95%)	20973 (34.26%)	20988 (34.28%)
O	393 (0.64%)	25922 (42.34%)	26458 (43.22%)	28063 (45.84%)	28195 (46.06%)
B	550 (0.90%)	6288 (10.27%)	3051 (4.98%)	5293 (8.65%)	5726 (9.35%)
N	n/a	1933 (3.16%)	563 (0.92%)	837 (1.37%)	1020 (1.67%)
T	56392 (92.12%)	6438 (10.52%)	10362 (16.93%)	6051 (9.88%)	5288 (8.64%)
G	27726	1188311	1003717	1272381	1309875
S	Not avail	13710904.75	13141046.21	12239980.85	10948011.57

Row **A** shows the number of blocks that were *A*lready optimal, i.e., those that cannot be optimized because they already consume the minimal amount of gas and ebso/syrup find bytecode with the same consumption. Row **O** contains the number of blocks that have been optimized and the found solution has been proven to be *O*ptimal, i.e., the one that consumes the minimum amount of gas needed to obtain the SFS provided. The solvers used are able to provide the best solution found until the timeout is reached. Row **B** contains the number of blocks that have been optimized into a *B*etter solution that consumes less gas but it is not shown to be the optimum. Row **N** shows the number of blocks that have *N*ot been optimized and not proven to be optimal, i.e., the solution found is the original one but there may exist a better one. Row **T** contains the number of blocks for which no model could be found when the *T*imeout was reached. Row **G** contains the accumulated *G*as savings for all optimized blocks. Importantly, the real savings would be larger if the optimized blocks are part of a loop and hence might be executed multiple times. Row **S** shows the time in *S*econds in which each setting analyzes all the blocks.

Let us first compare the results by ebso and our best results when using the portfolio of solvers in s-All. It is clear from the figures that syrup significantly outperforms ebso on the number of blocks handled (while ebso times out in 92.12 % of the blocks, we only timeout in 8.64 %) and on the overall gas gains (two orders of magnitude larger). For the analyzed blocks (i.e., those that do not timeout), the percentages of syrup for number of optimized into better blocks, into optimal blocks, and those proven to be already optimal, are much larger than those of ebso. We now discuss how the gains for the blocks that ebso can analyze compare to the gains by syrup. In particular, if missing part of the semantics of the uninterpreted instructions and the  bytecode significantly affects the gains. Out of 943 examples, where ebso found an optimization, in 46 cases syrup proved optimality w.r.t. the SFS and saved 348 gas but saved less gas than ebso (total 10514 gas). The source of this gain is the  bytecode: there are two blocks where ebso saves 5000 each, because it realizes that we read from a key in storage to then store the value back unchanged. As we discuss in Sect. 7, our framework naturally extends to handle this storage optimization. However, in nearly all of 393 cases, where ebso found an optimal solution—in 378 cases—syrup saves as much as ebso amounting to 2670 gas. That is, the additional semantics did not improve savings. Furthermore, in 43 cases out of 943, the semantics did impede ebso ’s performance so that syrup found a better result with 597 gas versus 440 of ebso. Therefore, we can conclude that syrup is far more scalable and precise than ebso, the cases in which syrup optimizes less than ebso are seldom and can be naturally handled in the future. Moreover, they are offset by the cases where syrup did find an optimization, whereas ebso did not.

Finally, we can see that MathSAT is the solver that shows the best performance: It proves optimality of 34.26 % and optimizes 54.49 % of the blocks (c.f. Sect. 5.3). Regarding analysis time, the global figure is not reported in [20]. In syrup, by accumulating the time of all four scenarios (s-X) and using the 900 s timeout of ebso, we analyze the whole data set in about 3042 h. We note that, by considering the solvers as a portfolio, we reduce the analysis time as when an optimal solution is found, the execution of the other two solvers is stopped. However, for the other cases, we take the highest time taken by the solvers as we need to know all solutions in order to keep the best one and provide an answer.

### Analysis of the Most Called Contracts with Gas Savings (setup Ii)

For our second setup, syrup produces the following results for the 46966 blocks of the 128 (most called) smart contracts:

**Fig. 5.**
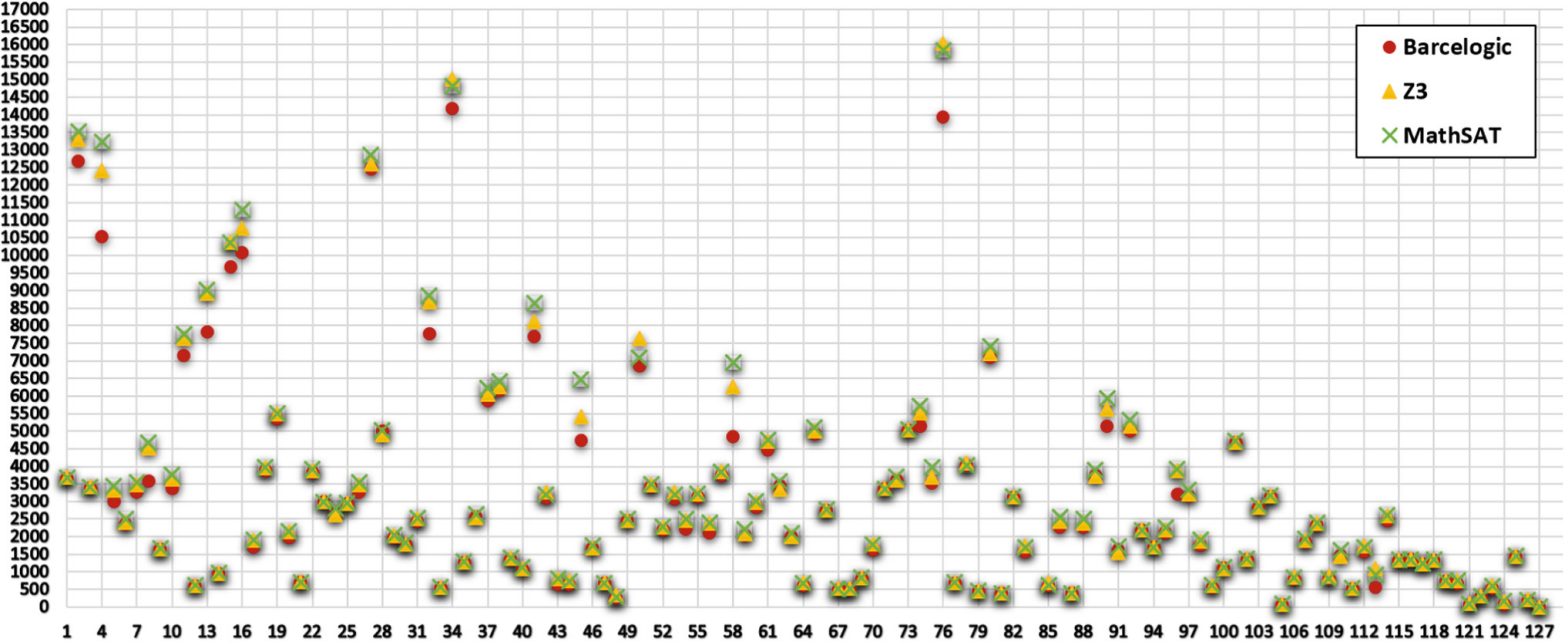
Gas saved per contract in the 128 most called smart contracts

**Table:** 

	s-Z3	s-Bar	s-OMS	s-All
A	30846 (65.68%)	30923 (65.84%)	30971 (65.94%)	30974 (65.95%)
O	13102 (27.9%)	13240 (28.19%)	13586 (28.93%)	13606 (28.97%)
B	933 (1.98%)	510 (1.09%)	746 (1.59%)	801 (1.71%)
N	695 (1.48%)	95 (0.2%)	295 (0.63%)	467 (0.99%)
T	1390 (2.96%)	2198 (4.68%)	1368 (2.91%)	1118 (2.38%)
G	438483	406086	437165	443248
S	2919830.35	2682469.58	2413612.39	2378446.26

As before, MathSAT is the solver that shows the best performance: It proves optimality of 65.94% and optimizes 30.52% of the blocks. The overall gas savings in **G** amount to 0.73% of the total gas which, assuming a uniform distribution of this saving among the contracts, amounts to around a million dollars from 2017 to 2019 (see Sect. 1 for details on this estimations). Moreover, we have calculated that the 64% of the saved gas is due to the simplification rules and the 36% to the Max-SMT optimization, which shows that both parts are highly relevant in our results. For this data set, we additionally display in Fig. 5 the amount of gas saved for each contract. The X-axis corresponds to each of the 128 analyzed contracts and the Y-axis corresponds to the amount of gas saved when using each solver. In general the gains obtained by the different solvers are quite aligned. On average, each contract saves 3425.65 units of gas using Z3, 3172.55 using Barcelogic and 3415.35 using MathSAT. However, we can observe that the gains are dispersed w.r.t. the mean, and there are big differences in the savings obtained for each of the contracts (the standard deviation is 2798.19 for Z3, 2664.05 for Barcelogic and 2889.01 for MathSAT). The biggest amount of gas optimized in all contracts is 18989 gas using Z3, 18704 using Barcelogic and 19205 using MathSAT. In the case of this contract, MathSAT optimizes 706 blocks out of 1910, and the highest amount of gas optimized is 162 though the most common amount of gas optimized is 3 (in 165 blocks). The highest amount of gas optimized per block in all contracts is 481. Finally, we have analyzed the impact of our optimization on the function transfer of the AirdropToken smart contract, that has been called around 520000 times. For this function, which has no loops, syrup saves 832 units of gas per call. From the number of calls per day (obtained from [2]), we estimate a total saving (just for this function) of 2815 $.

**Fig. 6.**
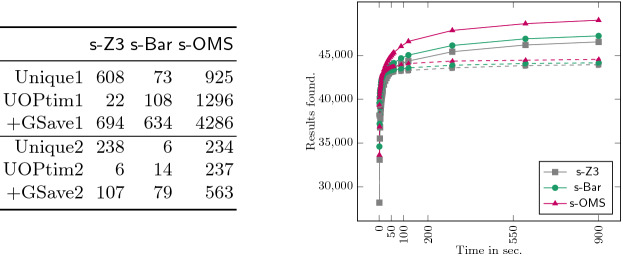
Comparison of SMT Solvers

### Comparison of SMT Solvers in Precision and Time

Figure 6 aims at providing some data to compare the accuracy and efficiency of the process using the three SMT solvers. The table to the left shows in: **Unique** the number of blocks that are uniquely optimized by the corresponding solver, in **UOptim** the number of blocks that are proven to be optimal uniquely by one solver, and **+GSave** the number of blocks for which one solver has strictly more gains that the others. The suffixes 1 and 2 refer to the data set in Sects. 5.1 and 5.2, resp., excluding all timeouts. In both data sets, MathSAT uniquely finds a result, uniquely shows the block optimal, or finds the best gain for the large majority. But clearly, in both data sets, every solver was needed to get the best possible solution in every category. The plot to the right of Fig. 6 displays the amount of blocks (Y-axis) that are solved in the corresponding amount of time (X-axis). Dashed lines correspond to data set 1 and plain lines to 2. We can see that for data set (i) within 10 s, nearly 89% of the results were found. For data set (ii) this is even more pronounced, after 10 s around 95% were found, with around 90% already being available after 1 s. The analysis shows that most results can be found very fast and thus our optimizer could be invoked during the compilation of a smart contract without adding a large overhead to compilation.

## Related Work

There are currently two automated approaches to gas optimization of Ethereum smart contracts. (i) First, the closest to ours is blockchain superoptimization [20], whose goal is the same as ours: find the gas-optimal block of code for each of the blocks in the CFG of the smart contract. While the approach of [20] would not be applicable within a compiler (e.g., it times out in 92.12 % of the blocks used in their experimental evaluation), our optimization tool performs very efficiently (e.g., we have seen that 89% of the blocks are optimized in less than 10 s). The reasons for our efficiency are indeed the fundamental differences with [20]: (1) we use the SFS to solve the optimization problem efficiently as a synthesis problem in which the semantic optimizations are carried out within the SFS part, (2) we do not encode the semantics of the arithmetic and bit-vector operations in the SMT problem, as [20] does, what allows us to express the problem using only existential quantification, (3) we use Max-SMT to solve the optimization problem. The basis for ebso is in [15], where the description of an encoding of unbounded superoptimization with the idea to shift the search for optimal program to the SMT solver is first found. (ii) Second, the system Gasol [5] incorporates also an automatic optimization for storage operations that consists in replacing accesses to the storage (i.e., bytecodes  and  ) by equivalent accesses to memory locations (i.e., bytecodes  and  ), when a static analysis identifies that it is sound and efficient doing such transformation. This optimization is not intra-block, as done in supercompilation, therefore it is not achievable by our approach as it involves modifying multiple blocks, and also requires an analysis that identifies the patterns and the soundness of the transformation. On the other hand, Gasol is not able to make the intra-block optimizations that we are achieving. Therefore, the optimizations in Gasol are orthogonal (and complementary) to those achievable by means of superoptimization.

There is work also focused on identifying gas expensive patterns: (1) the work in [9] identifies 7 expensive patterns on Solidity contracts and proposes optimizations for them. However, there is no tool in [9] that carries out these optimizations automatically; (2) The work in [10] identifies 24 anti-patterns, e.g. [  ] optimizes to  . Again, there is not automation and those patterns are manually identified. There is recent work that experimentally proves that the gas model for some EVM instructions is not correctly aligned with respect to the observed computational costs in real experiments [26], and that these misalignments can lead to gas-related attacks [22]. Our work is parametric on the gas model used, and new adjustments in the gas model of Ethereum are integrated in our optimizer by just updating the cost for the corresponding modified instructions in our implementation. Finally, the tool TOAST [8] also superoptimizes machine code. Although applied to different settings, the performance of syrup is significantly better and the notions of optimality used are different (sequence length and gas-usage respectively).

## Conclusions and Future Work

We have presented a novel method for gas super-optimization of smart contracts that combines symbolic execution with an effective Max-SMT encoding. Our focus is on the stack operations because these bytecode operations allow for multiple reorderings, simplifications, and cover the major part of the potential optimizations; while reading and/or writing on memory or storage can be seldom optimized (unless the same value is written, or read, consecutively). In spite of this, the same methodology we have formalized for the stack could be extended to optimize the memory and storage bytecode operations. Basically, the symbolic execution phase would extract a functional specification also for memory and for storage that would be analogous to our SFS and that could include storage-related optimizations (e.g., detecting unnecessary storage). The SMT encoding for these operations would be similar to ours but, for soundness, would have to maintain the order among the memory and storage accesses. It is part of our future work to implement also the super-optimizations for memory and storage and experimentally evaluate if there is significant gain. We also plan to extend the SMT encoding to include information gained from the original program such as the original cost. Currently, in roughly 0.05% of the blocks of Sect. 5.2, syrup synthesizes a more expensive solution.

## Footnotes

1 The data is taken from [3] using the gas spent by transactions and the average *gwei* and Ether exchange rate per day.

2 It is the most popular programming language for writing Ethereum smart contracts.

3 Up to Ethereum blockchain block number 7300000 until 2019-03-04 01:22:15 UTC.

4 Up to Ethereum blockchain block number 9193265 until 2019-12-31 23:59:45 UTC.
